# ﻿Editorial

**DOI:** 10.3897/zookeys.1252.153532

**Published:** 2025-09-19

**Authors:** Caroline S. Chaboo, Yoko Matsumura, Michael Schmitt

**Affiliations:** 1 University of Nebraska State Museum, W436 Nebraska Hall, Lincoln, Nebraska, 68588-0514, USA University of Nebraska State Museum Lincoln United States of America; 2 Systematic Entomology, Graduate School of Agriculture, Hokkaido University, Sapporo 060-8589, Japan Hokkaido University Sapporo Japan; 3 Unit Integrative Zoology, Department of Evolutionary Biology, University of Vienna, Djerassiplatz 1, 1030 Vienna, Austria University of Vienna Vienna Austria; 4 Universitaet Greifswald, Allgemeine & Systematische Zoologie Loitzer Str. 26, D-17489, Greifswald, Germany Universitaet Greifswald Greifswald Germany

This volume assembles ten research papers and one history article that emerged from presentations at the 11^th^ International Symposium on the Chrysomelidae, held in August 2024, at the 27^th^ International Congress of Entomology, in Kyoto, Japan. The volume continues the historic tradition of special publications “Research on Chrysomelidae” that started in 2008 with RoC 1, published by Brill Academic Publishers, from RoC 3 as Special Issues of ZooKeys, published by Pensoft (Sofia). These special volumes were the idea of Pierre Jolivet (1922–2020), and the series has continued over the years to today’s RoC 10, from various meetings with different Chrysomelidae symposia organizers and volume editors (Table 1).

**Table 1.** Summary of special volumes on Chrysomelidae.

Volume	Year	Editors	Meeting	City/Country
First International Symposium on the Chrysomelidae. Entomography 3: 371–503	1985	David G. Furth & Terry N. Seeno	1^st^ International Symposium on the Chrysomelidae. 1984	Hamburg, Germany
Biology of Chrysomelidae. Kluwer Academic Publ., Dordrecht etc.	1988	Pierre Jolivet, Eduard Petitpierre, & Ting H. Hsiao		
Second International Symposium on the Chrysomelidae. Entomography 6: 343–552	1989	David G. Furth & Terry N. Seeno	2nd International Symposium on the Chrysomelidae. 1988	Vancouver, Canada
Proceedings of 3^rd^ International Symposium on the Chrysomelidae. Backhuis, Leiden	1994	David G. Furth	3^rd^ International Symposium on the Chrysomelidae. 1992	Beijing, China
Novel Aspects of the Biology of Chrysomelidae. Kluwer Academic Publ., Dordrecht etc.	1994	Pierre H. Jolivet, Michael L. Cox, & Eduard Petitpierre		
Chrysomelidae Biology, 3 vols, SPB Academic Publ., Amsterdam	1996	Pierre H.A. Jolivet & Michael L. Cox		
Proceedings of the Fourth International Symposium on the Chrysomelidae. Atti Museo Regionale di Scienze Naturali Torino	1998	Maurizio Biondi, Mauro Daccordi, & David G. Furth	4^th^ International Symposium on the Chrysomelidae. 1996	Florence, Italy
Advances in Chrysomelidae Biology 1. Backhuis Publ., Leiden	1999	Michael L. Cox		
Special Topics in Leaf Beetle Biology – Proceedings of the Fifth International Symposium on the Chrysomelidae	2003	David G. Furth	5^th^ International Symposium on the Chrysomelidae. 2000	Iguassu, Brazil
New Developments in the Biology of Chrysomelidae. SPB Academic Publ., The Hague	2004	Pierre Jolivet, Jorge A. Santiago-Blay, & Michael Schmitt		
Proceedings of the 6^th^ International Symposium on the Chrysomelidae. Bonner zoologische Beiträge 54(4): 173–312 (2005)	2006	Michael Schmitt	6^th^ International Symposium on the Chrysomelidae. 2004	Bonn, Germany
Research on Chrysomelidae (RoC) 1. Brill Publ., Leiden - Boston	2008	Pierre Jolivet, Jorge A. Santiago-Blay, & Michael Schmitt		
Research on Chrysomelidae (RoC) 2. Brill Publ., Leiden - Boston	2009	Pierre Jolivet, Jorge A. Santiago-Blay, & Michael Schmitt	7^th^ International Symposium on the Chrysomelidae. 2008	Durban, South Africa
Research on Chrysomelidae (RoC) 3. ZooKeys 157: 1–179	2011	Pierre Jolivet, Jorge A. Santiago-Blay, & Michael Schmitt	1^st^ European Symposium on the Chrysomelidae 2010	Budapest, Hungary
Research on Chrysomelidae (RoC) 4. ZooKeys 332: 1–231	2013	Pierre Jolivet, Jorge A. Santiago-Blay, & Michael Schmitt	8^th^ International Symposium on the Chrysomelidae 2012	Daegu, South Korea
Research on Chrysomelidae (RoC) 5. ZooKeys 547: 1–203	2015	Pierre Jolivet, Jorge A. Santiago-Blay, & Michael Schmitt	2^nd^ European Symposium on the Chrysomelidae 2014	York, England, UK
Research on Chrysomelidae (RoC) 6. ZooKeys 597: 1–99	2016	Pierre Jolivet, Jorge A. Santiago-Blay, & Michael Schmitt		
Research on Chrysomelidae (RoC) 7. ZooKeys 720: 1–137	2017	Caroline S. Chaboo & Michael Schmitt	9^th^ International Symposium on the Chrysomelidae 2016	Orlando, FL, USA
Research on Chrysomelidae (RoC) 8. ZooKeys 856: 1–196	2019	Caroline S. Chaboo, Michael Schmitt, & Maurizio Biondi	3^rd^ European Symposium on the Chrysomelidae 2018	Naples, Italy
Research on Chrysomelidae (RoC) 9. ZooKeys 1177: 1–258	2023	Caroline S. Chaboo & Michael Schmitt	10^th^ International Symposium on the Chrysomelidae 2022	Helsinki, Finland
Research on Chrysomelidae (RoC) 10. ZooKeys 1–233	2025	Caroline S. Chaboo, Yoko Matsumura, & Michael Schmitt	11^th^ International Symposium on the Chrysomelidae 2024	Kyoto, Japan

The present volume brings together the latest research in biology and systematics from diverse authors and research teams in the Americas, China, Europe, Japan, and Taiwan. The contributors included new students of Chrysomelidae, established experts, and senior retirees. The papers included cover history, taxonomy, morphology, ecology, and pest management and reflect the diversity of contemporary research in Chrysomelidae.

The ICE Kyoto was held in Japan for the first time after 44 years (XVI ICE 1980) and was the biggest entomological conference after the COVID pandemic, providing a brilliant chance for Japanese students/ young postdocs to meet international entomologists. Indeed, the fact that one-fourth of the participants were from Japan (1,084 out of a total 4,041 registered participants) reflects the high expec­tations of Japanesse scientists. A commemorative review book composed of several chapters, including all major topics dealt with in ICE 2024, is planned and is now under a review process in Japan. Regarding our symposium, we received four of 15 talks and one poster presentation from Japanese students/ young postdocs (on top of these, three more presentations were given by Japanese scientists). The symposium provided the Japanese students/ young postdocs with an invaluable and unforgettable opportunity to meet international fellows and experts.

The Facebook group of chrysomelid enthusiasts, Chrysomelidae Forum, comprises today 4,700 members (from 426 members in 2017) and has become a daily forum for announcing news, especially new publications, and obtaining identifications for Chrysomelidae. The Chrysomela newsletters (vol. 56 in 2025, editor CS Chaboo) are published informally from time to time. Our next international meetings will be at the 13^th^ European Congress of Entomology (ECE), Tours, France in July 2026 and the 28^th^ International Congress of Entomology, Cape Town, South Africa in July 2028. We hope many researchers will join us to meet in person and update our community on new methods and approaches in Chrysomelidae research.

**Figures 1–3.**
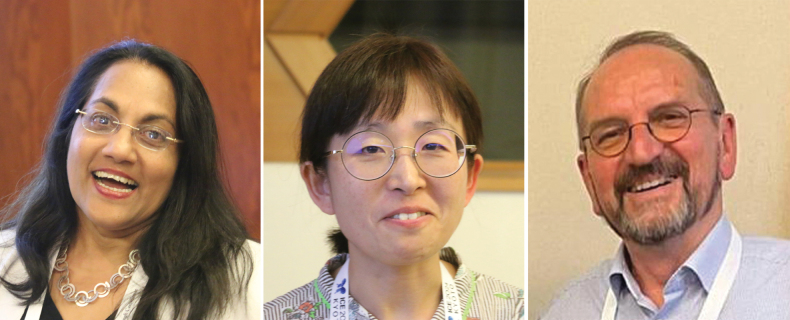
Editors of RoC 10 **1** Caroline S Chaboo **2** Yoko Matsumura **3** Michael Schmitt.

After co-editing 12 volumes on Chrysomelidae, among them ten volumes of RoC, editor “Theo” Michael Schmitt steps down from the editorial board, hoping that younger motivated editors continue the series “Research on Chrysomelidae”.

The editors are grateful to Yordanka Banalieva, Lyubomir Penev, and all the other friendly staff at Pensoft for a smooth and joyful co-operation.

## ﻿Additional information

### Author ORCIDs

Caroline S. Chaboo https://orcid.org/0000-0002-6983-8042

Yoko Matsumura https://orcid.org/0000-0002-3438-2161

Michael Schmitt https://orcid.org/0000-0001-7377-3643

### Data availability

All of the data that support the findings of this study are available in the main text.

